# Hydrothermal synthesis and methylene blue adsorption performance of novel 3D hierarchical Li_2_Si_2_O_5_ hydrate particles

**DOI:** 10.1038/s41598-020-62462-5

**Published:** 2020-03-26

**Authors:** Hui Zhang, Jinxiao Wang, Jianfeng Yang

**Affiliations:** State Key Laboratory for Mechanical Behavior of Materials, Xi’an Jiaotong University, Xi’an, 710049 China

**Keywords:** Environmental sciences, Ceramics

## Abstract

Li_2_Si_2_O_5_ are generally obtained in form of granules with unavoidable impurities including Li_2_SiO_3_ and SiO_2_. Here, we demonstrated a facile hydrothermal route to synthesize novel 3D hierarchical Li_2_Si_2_O_5_ hydrate hollow flower-like microstructures assembled by rod subunits with high purity. The crystal growth was accomplished by complete transformation from poorly crystallized metastable phases formed in the initial stage including Li_2_SiO_3_, SiO_2_ and various Li_2_Si_2_O_5_ hydrate species to Li_2_Si_2_O_5_ hydrate rods. The transformation over many times gave a sustainable high chemical potential to direct the anisotropic growth of Li_2_Si_2_O_5_ hydrate rods with large aspect ratios. Besides, the variation of Li/Si molar ratios confirmed that Li_2_Si_2_O_5_ hydrate rods were obtained only at Li/Si = 1. The perfection and aspect ratio of the rods could be controlled very well by adjusting the hydrothermal temperatures and precursor concentrations. Some new points about obtaining pure phase and anisotropic morphology were discussed, including careful selection of precursors and synthetic method. The obtained novel 3D Li_2_Si_2_O_5_ hydrate structures exhibited a characteristic of mesoporous material and had an excellent adsorption capability of methylene blue with high adsorption amount of 49.42 mg·g^−1^ and color removal of 98.85%, indicating the potential use in wastewater treatment.

## Introduction

Functional materials particularly with 3D hierarchical structures assembled by 1D or 2D building blocks have attracted rapidly increasing attention due to their amazing versatility in physical-chemistry and biological field^1–4^. Their performances are closely related to the phase composition, crystal size, shape and the assembly model of building units that is determined by complex synthetic parameters^5–8^. It is still an urgent need to develop effective methods to obtain the 3D hierarchical structures with pure phase and specific morphologies when scientists are devoted to studying these materials’ characteristics and broadening the fields of their capabilities.

Among these materials, lithium disilicate (Li_2_Si_2_O_5_), with unique sandwich crystal structure capable of Li-ion transportation in dimensional direction among the corrugated [SiO_4_] layers, have played distinguishable roles in fundamental studies and technical applications owing to its superior properties such as chemical stability, high strength, good thermal stability, and its unique biocompatibility for applications in dental materials and adsorption field^9–16^. Some methods have been used to synthetize single-phase Li_2_Si_2_O_5_ powders with desirable shape, but unfortunately, mixture products among bulk-like Li_2_SiO_3_, Li_2_Si_2_O_5_ and SiO_2_, are often inevitably generated via solid-state reaction^17^, combustion approaches^18^ and sol-gel method^19^. The residual Li_2_SiO_3_ produced by these methods were too stubborn to transform into Li_2_Si_2_O_5_ even using excessive molar ratios of Si/Li > 1 (stoichiometric ratio of Li_2_Si_2_O_5_ = 1), due to Li_2_SiO_3_ aggregates can be obtained easily at low temperature (~20 °C) and exhibit excellent thermal stability even after calcination at 900 °C^20^. Thus, the incomplete solid-phase reaction attributed to the dynamic deficiency of solid-state diffusion with a slow rate at later period, is the emphatic shortcoming for these routes.

Not only the challenge in purity, but also the anisotropic crystal growth of Li_2_Si_2_O_5_ is out of control due to the fast reaction rate related to the characteristic nature of Li^+^, resulting in irregular bulk shape at the initial stage of reaction, as evidenced by the above routes. To achieve 1D growth, the higher chemical potential of precursor compared with that of final crystal product is generally needed under non surfactant system, and sufficient transmission is also demanded strongly to achieve a complete reaction^21–23^, this means that the precursor for growth of 1D Li_2_Si_2_O_5_ crystals must possess appropriate dissolving rate and effective contact with mediums. Therefore, the insoluble crystal phase precursor, such as the crystallized Li_2_SiO_3_ aggregates must to be avoided.

Recently, researches indicate that lithium silicates with 3D hierarchical assembly structures exhibit enhanced adsorption and photoefficiency, and have potential in wastewater treatment such as dye adsorption^24,25^. A mild hydrothermal method has been successfully employed to prepare pure 2D sheet-like Li_2_Si_2_O_5_ by using LiNO_3_, silicic acid and NaOH with molar ratios of Si/Li ≥ 2^26,27^. *Alemi et al*. have also obtained 3D bundle-like lithium sodium disilicate (silinaite) assembly with excellent photoluminescence performance by the hydrothermal method using Li_2_SO_4_·H_2_O, silicic acid and NaOH with Si/Li = 1^28^. It is a pity that the additional Si source was needed to fabricate pure phase of Li_2_Si_2_O_5_ products. What is more, up to now, little success has been achieved in constructing hierarchical Li_2_Si_2_O_5_ structures with pure phase and anisotropic building blocks. This is because that Li^+^ with small radii shows markedly difference compared with other metal ions. Meanwhile, to our knowledge, almost all of the above publications just focus on the preparation of a defined Li_2_Si_2_O_5_ structure, it is also a pity that no light has been cast on the morphological evolution and growth mechanism of Li_2_Si_2_O_5_ structures.

Here, we develop a facile hydrothermal route to achieve novel hierarchical 3D hollow Li_2_Si_2_O_5_ hydrate microstructures assembled by rod-like single-crystals using LiOH·H_2_O and TEOS as precursor materials. It demonstrates that molar ratio of Li/Si = 1 can also been used to obtain pure Li_2_Si_2_O_5_ hydrate crystals, a complex process involving nucleation of nanoparticles, agglomeration and *in-situ* crystallization, and inside-out Ostwald ripening is put forward for the formation and morphology evolution of the 3D Li_2_Si_2_O_5_ hydrate microstructures. The metastable aggregates resulted from fast reaction at the initial period, are regarded as the precursors for the subsequent anisotropic crystal growth. Besides, influences of Li/Si molar ratios, temperatures, precursor concentrations on phase composition, morphology and particle size are systematically studied, new sight in the formation mechanism is discussed detailedly. Moreover, the excellent methylene blue adsorption performance is selected to evaluate the capability of the Li_2_Si_2_O_5_ hydrate products for dye wastewater treatment. This information will be useful and pioneering for further research and other practical applications of the novel structures in lithium silicate series.

## Experimental

### Synthesis of materials

The reagents used in the experiments were of analytical grade without further purification. In a typical procedure, 1.68 g of LiOH·H_2_O was magnetically stirred together with 80 mL of deionized water in a glass beaker until completely dissolved, resulting in a colorless transparent solution with concentration of 0.5 M. Then, 8.335 g of TEOS was dropped into the solution with stoichiometric molar ratio of Li/Si = 1. After vigorously stirring for 30 min, the mixed solution was transferred into a 100 mL Teflon-lined autoclave and maintained at 180 °C with soaking time of 1 to 48 h to investigate the morphology evolution of intermediates. The as-obtained white products were collected by centrifugation, washed several times with alcohol, and then dried in air at 80 °C for 24 h. To investigate the effect on morphology, phase composition and size of products, we varied the Li/Si molar ratio (Li/Si = 0.5, 0.8, 1, and 1.5), hydrothermal temperature (100, 120, 150, and 180 °C) and precursor concentration (0.1, 0.2, 0.3, 0.4, 0.5 M).

### Characterization

The crystalline phase of the products was characterized by powder X-ray diffraction (XRD; Empyrean, PANalytical, the Netherlands) using Cu Kα radiation (λ = 1.5418 Å) at a scanning rate of 0.05° s^−1^ operating at 40 kV and 40 mA. The morphologies of the products were obtained by a thermal field emission scanning electron microscope (FE-SEM, S-4800, Hitachi, Tokyo, Japan) with an accelerating voltage of 10 kV. High resolution transmission electron microscopy (HRTEM) and selected area electron diffraction (SAED) patterns of the products were obtained on a transmission electron microscope (JEOL, JEM-2100, Japan) operated at 200 kV. The Brunauer-Emmett-Teller (BET) parameters: specific surface area, pore size and volume of products, were measured from the N_2_ adsorption isotherm by using 3H-2000 automatic specific surface area analyzer (Beijing, China).

### Adsorption performance experiments

The adsorption kinetic experiments were conducted by adding 20 mg of products into 50 mL of methylene blue aqueous solution (20 ppm (mg·L^−1^)) at room temperature with continuous stirring at 650 rpm. The samples were withdrawn at predetermined time intervals until the adsorption reached equilibrium, and the adsorbents were centrifuged at 5000 rpm for 5 min. Residual methylene blue concentration in the supernatant was detected by UV-vis spectrophotometer (UV-1800PC, Shanghai Mapada Instruments Co., Ltd., China) at *λ*_max_ = 664 nm, corresponding to the maximum absorption wavelengths of the methylene blue. The adsorption of methylene blue dyes at any time (*Q*_*t*_ (mg·g^−1^)) and color removal (%) were calculated by the following equations, respectively:

1 $${{Q}}_{{t}}=({{C}}_{0}-{{C}}_{{t}})\,{V}/{W}$$

2 $${Color}\,{removal}=({{C}}_{{0}}-{{C}}_{{t}})/{{C}}_{0}\times 100 \% ;$$

where *C*_0_ (mg·L^−1^) is the initial methylene blue concentration, *C*_*t*_ (mg·L^−1^) is the concentration of methylene blue solution at time t, *V* is the methylene blue solution volume (L), and *W* is the weight of the absorbent used (g).

## Results and discussion

### Synthesis of Li_2_Si_2_O_5_ hydrate

**Figure 1:**
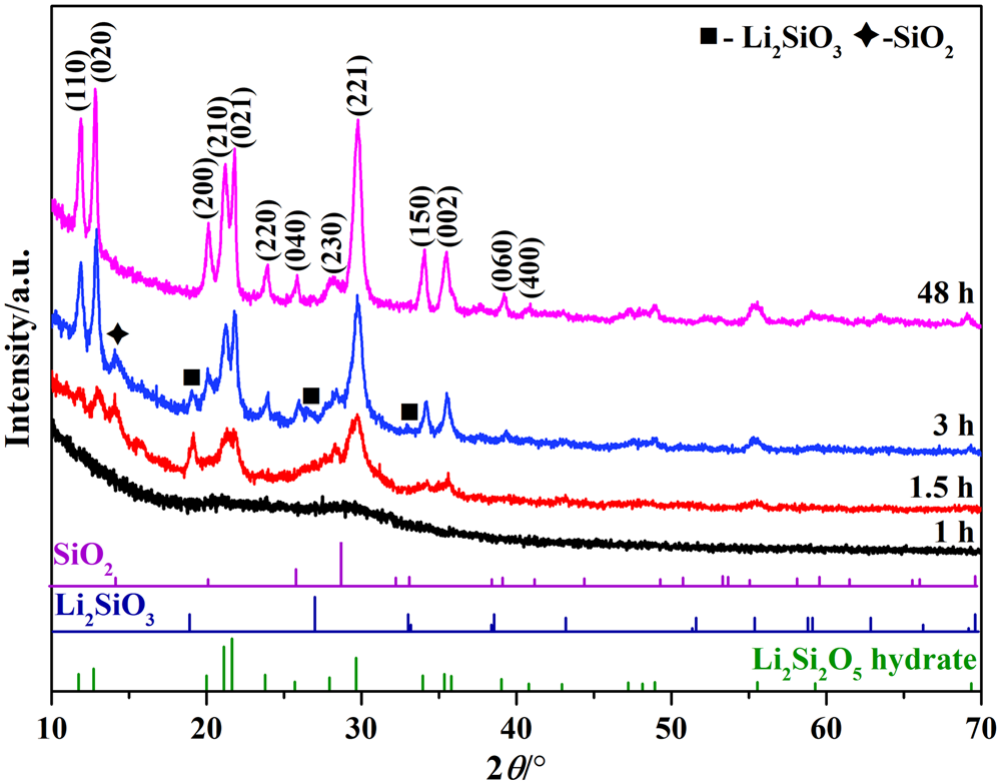
The XRD patterns of the intermediate products prepared at different hydrothermal stages under Li/Si = 1.

Figure 1 shows the developmental evolution of crystalline phases of the intermediate products at different hydrothermal stages under Li/Si = 1. At 1 h, only several unapparent diffraction peaks in the XRD pattern could be found and indexed to Li_2_SiO_3_ (JCPDS No. 30-0766) and Li_2_Si_2_O_5_ hydrate (Li_2_Si_2_O_5_·2H_2_O, JCPDS No. 33-0816), implying a mixture product with extremely poor crystallinity. Slightly extending time to 1.5 h, the peak intensity of both of Li_2_SiO_3_ and Li_2_Si_2_O_5_ hydrate increased relatively, indicating the mixture product still possessed relative poor crystallinity. There was also poor-crystallized SiO_2_ formed at this stage. Prolonging the time to 3 h, the peak intensity of SiO_2_ did not change obviously, while Li_2_Si_2_O_5_ hydrate peaks intensely increased with synchronous decrease of Li_2_SiO_3_. After 48 h, both of Li_2_SiO_3_ and SiO_2_ disappeared, and only well-crystallized Li_2_Si_2_O_5_ hydrate was found as the final product, demonstrating the complete transformation from poor-crystallized Li_2_SiO_3_ and SiO_2_ into Li_2_Si_2_O_5_ hydrate.

**Figure 2:**
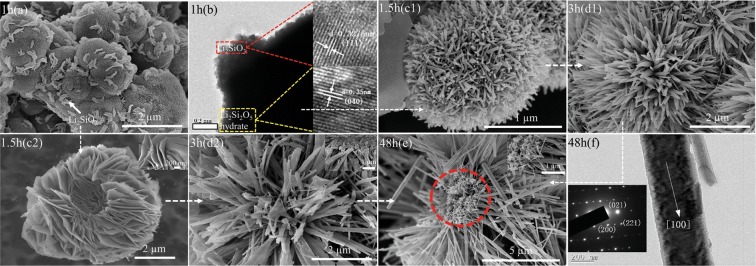
The morphological evolution of the intermediate products at different stages: 1 h (**a**) FE-SEM and (**b**) TEM and HRTEM; 1.5 h (**c1**,**c2**) FE-SEM; 3 h **(d1**,**d2**) FE-SEM; 48 h (**e**) FE-SEM and (**f**) TEM and corresponding SAED pattern.

Figure 2 presents the corresponding morphological evolution of the intermediate products. At the first 1 h (Fig. 2a), solid microspheres with an average diameter of 1.5 µm were obtained and composed of numerous nanoparticles. Adhered on their surfaces were some randomly distributed crescent-like structures. TEM and HRTEM images (Fig. 2b) demonstrated that the solid microspheres and surface crescent-like structures could be well assigned to Li_2_Si_2_O_5_ hydrate by a lattice spacing of 0.35 nm corresponding to (040) plane and Li_2_SiO_3_ by a lattice spacing of 0.3275 nm indexed to (111) plane, respectively. This result was well matched to the XRD analysis (Fig. 1(1h)). When the reaction time was slightly prolonged to 1.5 h, with almost no size changing, the solid Li_2_Si_2_O_5_ hydrate microspheres were crystallized into porous ones which consisted of numerous loosely packed sticks (Fig. 2(c1)), suggesting an *in situ* crystallization process. Simultaneously, the Li_2_SiO_3_ crescent-like structures grew into hollow peony-like microspheres that comprised of flexible petal-like nanosheets with a thickness of ~20 nm (Fig. 2(c2)). With a longer process of 3 h, Li_2_Si_2_O_5_ hydrate porous microspheres developed into urchin-like ones with a swelling diameter of about 4 µm, which were constructed by long nanorods (Fig. 2(d1)). However, the peony-like structures disappeared and Li_2_SiO_3_ nanosheet petals split into linked rods (Fig. 2(d2)), suggesting that the transformation of Li_2_SiO_3_-to-Li_2_Si_2_O_5_ hydrate took place, also confirmed by the XRD result (Fig. 1(3h)). Their broken roots were connected with fragments (the inset in Fig. 2(d2)), indicating that the transformation here had not started yet. By the way, we did not observe any SiO_2_ existed in these two stages (1.5 and 3 h), which might be washed away during the sample preparation for FE-SEM due to their small size and slight amount. After 48 h (Fig. 2e), all of the fragments disappeared, and 3D hollow flower-like assembly of prism-like rods were solely developed, in consistent with the XRD result (Fig. 1(48h)). It was noted that all of the rods were organized toward a common center essentially composed of tiny sticks shown in the red circle. The mean length and diameter of the rods were about 7 µm and 300 nm, respectively, indicating a high aspect ratio of more than 20 that implied the maintenance of the growth anisotropy during the hydrothermal processes. TEM image (Fig. 2f) showed that there were mesoporous pores on the surface of the rods, and the corresponding SAED pattern demonstrated that the rod possessed a well-crystallized single-crystal characteristic with a preference growth direction along [100].

### Formation mechanism

**Figure 3:**
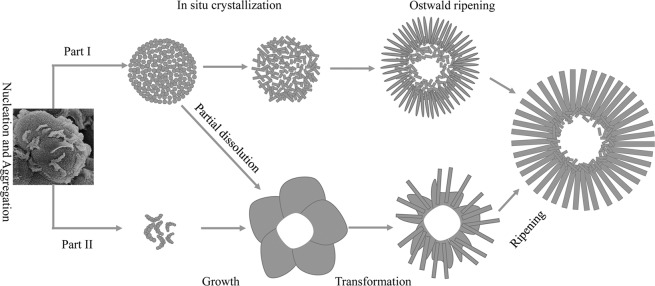
The formation mechanism of the novel 3D Li_2_Si_2_O_5_ hydrate rod assembly.

On the basis of the above results, a possible mechanism for the formation of the novel 3D Li_2_Si_2_O_5_ hydrate rod assembly has been schemed in Fig. 3, and the detailed processes including two concurrent Parts for the shape evolution are illustrated as follows.

At the very beginning (1 h), silicic acid molecules, produced rapidly via fast hydrolysis of TEOS in alkaline environment at high temperature, quickly bonded with ambient Li^+^ to form Li_2_Si_2_O_5_ hydrate crystallitic nanoparticles (Eq. 1) driven by their initial high supersaturation degree. For reducing the high surface energy, these freshly formed crystallites spontaneously aggregated with each other to form larger poorly crystallized solid microspheres. After the rapid reaction period, Li^+^-rich micro regions were brought due to the insufficient supply of silicic acid caused by the delayed hydrolysis of TEOS, promoting the heterogeneous nucleation of Li_2_SiO_3_ (Eq. 2)^20,28^. These nuclei subsequently self-organized into poor-crystallized crescent-like nanostructures on the surfaces of Li_2_Si_2_O_5_ hydrate solid microspheres to minimize the surface energy. With the reaction continuing (1.5 h), the supersaturation of solution fell to some extent, the poorly crystallized Li_2_Si_2_O_5_ hydrate became thermodynamically metastable due to thermal nonequilibrium, resulting in fast dissolution of the crystallites. This in turn instantaneously increased supersaturation of Li^+^ and silicic acid molecules in the whole body of microspheres, leading to an *in situ* nucleation and growth of Li_2_Si_2_O_5_ hydrate (Part I in Fig. 3). But unlike a uniform ion reaction system, the dissolution process needed a certain amount of time, the newly formed Li_2_Si_2_O_5_ nuclei could consume the continuously dissolved ions to form elongated shapes before the ion concentration decreased to a critical value, even though the *in situ* crystallized Li_2_Si_2_O_5_ hydrate sticks in the porous microspheres still possessed low crystallinity in the fast dissolution process. At the same time, part of the dissolved ions would diffuse to the outer surface, benefitting the peony-like growth of Li_2_SiO_3_ (Part II in Fig. 3) in the sustained Li^+^-rich area due to the faster diffusion rate of Li^+^ than that of silicic acid molecules. As a result, the porous Li_2_Si_2_O_5_ hydrate microspheres and peony-like Li_2_SiO_3_ were formed and still controlled by the kinetic factor of supersaturation. While the growth of Li_2_SiO_3_ gave rise to the surplus of silicic acid, providing a chance to precipitate crystalline SiO_2_ by the condensation of the silicic acid from the dissolution of Li_2_Si_2_O_5_ hydrate crystallites or the hydrolysis of TEOS. Because of the faster nucleation rate and the production of more sticks with smaller sizes, the sticks with low-crystallinity located in the interior had higher solubility in terms of higher surface energy associated with larger curvature than that located in the outer surface. Thus the sticks located in the interior dissolved preferentially to deposit on the corresponding exterior sticks with identical crystallographic directions over the similar surface symmetries^29^ and a nearly zero-kinetic barrier^30^. This is an inside-out Ostwald ripening process (3–48 h) thermodynamically controlled by the dissolution rate of the sticks. Compared with their amorphous species, the sticks with certain crystallinity were capable of a slower dissolution rate to facilitate a continuously high chemical potential level to direct the one-dimensional growth of the Li_2_Si_2_O_5_ hydrate rods.

3 $$2\,{{\rm{Li}}}^{+}+2\,{{\rm{OH}}}^{-}+2\,{{\rm{SiO}}}_{2}\cdot {{\rm{H}}}_{2}{\rm{O}}={{\rm{Li}}}_{2}{{\rm{Si}}}_{2}{{\rm{O}}}_{5}\cdot 2{{\rm{H}}}_{2}{\rm{O}}+{{\rm{H}}}_{2}{\rm{O}}$$

4 $$2\,{{\rm{Li}}}^{+}+{{\rm{SiO}}}_{2}\cdot {{\rm{H}}}_{2}{\rm{O}}+2\,{{\rm{OH}}}^{-}={{\rm{Li}}}_{2}{{\rm{SiO}}}_{3}+2{{\rm{H}}}_{2}{\rm{O}}$$

**Figure 4:**
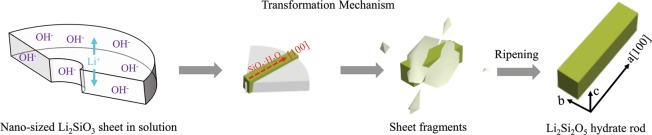
Transformation mechanism of Li_2_SiO_3_- Li_2_Si_2_O_5_ hydrate.

Accompanied with the dissolution of the Li_2_Si_2_O_5_ hydrate sticks, part of silicic acid molecules diffused to the surface of the Li_2_SiO_3_ nanosheets, leading to the incipient transformation of Li_2_SiO_3_-to-Li_2_Si_2_O_5_ hydrate. Right after the silicic acid bonded with the surface, the nanosheets then split and their derived products were formed in a similar shape of the nearly radial morphology (Part II in Fig. 3), agreed with *Huang et al*.’s report that elucidated the thickness of the Li_2_Si_2_O_5_ hydrate rods increased through sacrificing the width of the Li_2_SiO_3_ nanosheets^31^. This transformation process was described clearly in Fig. 4, and also enhanced by the dissolution of poor-crystallized SiO_2_ and surplus silicic acid. Li_2_Si_2_O_5_ hydrate nucleated within the Li_2_SiO_3_ nanosheet by way of that Li^+^ migrated in the interlayer structure to react with silicic acid molecules arrayed at the plane dependent upon the Li^+^ sites. Evidently, Li_2_Si_2_O_5_ hydrate grew in this manner and inherited the hollow and radial architecture of Li_2_SiO_3_ to evolve into the final rod assembly structure by the follow-up ripening process. Significantly in here, the poor-crystallized Li_2_SiO_3_ nanosheets possessed an ultrathin structure (~20 nm in thickness) and were thermodynamically metastable in the hydrothermal solution, as well the contiguous silicic acid molecules provided reaction dynamic support for their transformation to the final stable Li_2_Si_2_O_5_ hydrate. Therefore, structural Li^+^ could migrate in the ultrathin interlayer avoiding the long-range binding effect from the network structure itself, and easily react with silicic acid diffused to the surface of nanosheets achieving the complete Li_2_SiO_3_-to-Li_2_Si_2_O_5_ hydrate transformation.

Obviously, in comparison to other related reports^18,19,26,27^, the acquisition of high-purity Li_2_Si_2_O_5_ hydrate declared the significant importance in selecting LiOH·H_2_O and TEOS as precursors with Li/Si = 1 that avoided a common ion effect of Na^+^ replacing Li^+^ to percolate into [SiO_4_] network structure that eventually induced Li_2_SiO_3_ residues. It also demonstrated that in our work, the thermodynamically metastable Li_2_SiO_3_ nanosheets were the prerequisite for the complete Li_2_SiO_3_-to-Li_2_Si_2_O_5_ hydrate transformation at Li/Si = 1. Furthermore, poorly crystallized intermediates (including Li_2_SiO_3_ nanosheets, SiO_2_, and Li_2_Si_2_O_5_ hydrate nanoparticles) possessed a certain dissolution rate to ensure a continuously high chemical potential for anisotropic crystal growth, and were finally transformed completely into Li_2_Si_2_O_5_ hydrate crystals.

### Effect of synthesis parameters on products

**Figure 5:**
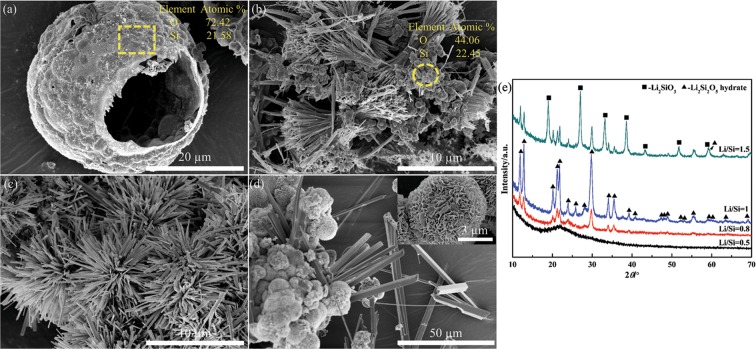
The FE-SEM images and corresponding XRD patterns of Li_2_Si_2_O_5_ products with different molar ratios synthesized at 180 °C for 48 h: (**a**) Li/Si = 0.5, (**b**) 0.8, (**c**) 1 and (**d**) 1.5; (**e**) corresponding XRD patterns.

Figure 5 shows the FE-SEM images and corresponding XRD patterns of the Li_2_Si_2_O_5_ products with different molar ratios synthesized at 180 °C for 48 h. For Li/Si = 0.5, it could be observed that hollow spheres with a mean size of about 20 µm were built by plenty of small particles, consisted of an amorphous mixture of SiO_2_ (Fig. 5(e) XRD) and Li_2_Si_2_O_5_ hydrate (Fig. 5(a)) confirmed by Si/O molar ratio near 2/7 through EDS analysis. Increasing the Li/Si molar ratio to 0.8, dumbbell-like Li_2_Si_2_O_5_ hydrate brushes were formed comprising numerous nanowires with an average length of about 10 µm, and adhered with many nanopartical SiO_2_ aggregations that were detected by EDS with the Si/O molar ratio near 1/2 (Fig. 5(b)). For Li/Si = 1.5, compared to Li/Si = 1 (Fig. 5(c)) as mentioned above, Li_2_Si_2_O_5_ hydrate rods became larger with length of greater than 20 µm, and were scattered intricately around the large peony-like Li_2_SiO_3_ particles assembled by nanosheets (Fig. 5(d)). The peak intensity of Li_2_Si_2_O_5_ hydrate increased remarkably as the Li/Si molar ratio increased from 0.8 to 1, indicating good-crystallinity (Fig. 5(e) XRD). But it decreased sharply when the Li/Si molar ratio increased to 1.5 due to the formation of well-crystallized peony-like Li_2_SiO_3_.

The considerable difference in morphologies of Li/Si = 0.5, 0.8 and 1 highlighted the crucial role of the Li/Si molar ratio. As is known, a higher chemical potential formed by OH^−^ is preferable for the growth of nanowires or nanorods^32,33^. Thus, when we used the stoichiometric ratio of Li/Si = 1 which produced higher OH^−^ ion concentrations, the nanorod morphology was obtained. Slight decrease in OH^−^ ion concentration (Li/Si = 0.8), crystal growth yielded namiwires. Further decrease OH^−^ ion concentration (Li/Si = 0.5) gave an amorphous hollow sphere. The results of evolution process based on molar ratios of Li/Si ≤ 1 was entirely consistent with the report^34^. But regarding Li/Si = 1.5, an entirely different process was established. Presumably, the Li/Si = 1.5 was close to the stoichiometric ratio (Li/Si = 2) of Li_2_SiO_3_, so peony-like Li_2_SiO_3_ formed earlier than rod-like Li_2_Si_2_O_5_ hydrate was in agreement with the nucleation kinetics calculation that Li_2_SiO_3_ has a lower activation energy than Li_2_Si_2_O_5_ phase^35^. After the consumption of precursors, rod-like Li_2_Si_2_O_5_ hydrate crystals was formed due to the achievement of both Li/Si molar ratio and supersaturation, resulting in a mixture of Li_2_SiO_3_ nanosheet flower and Li_2_Si_2_O_5_ hydrate rods.

**Figure 6:**
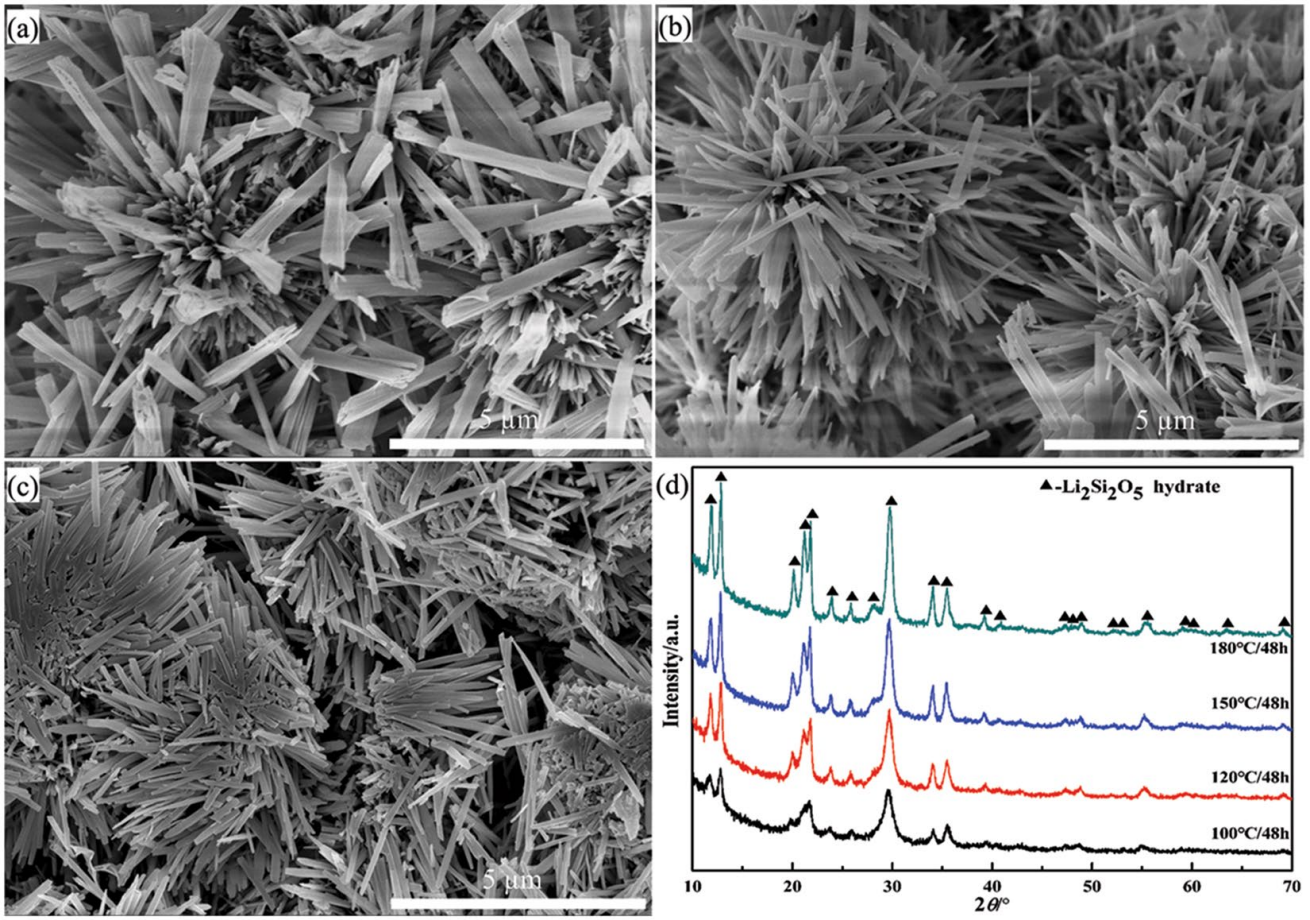
The FE-SEM images and XRD patterns of Li_2_Si_2_O_5_ hydrate products obtained at different hydrothermal temperatures whilst keeping Li/Si = 1: (**a**) 100, (**b**) 120 and (**c**) 150 °C; (**d**) XRD patterns.

Figure 6 shows the FE-SEM images and XRD patterns of Li_2_Si_2_O_5_ hydrate products obtained at different hydrothermal temperatures whilst keeping the Li/Si = 1. At 100 °C, flower-like structures constructed by layered rods were only obtained with a low crystallinity and a characteristic length of ~2 µm and width of ~400 nm. Elevating the temperature from 120 to 150 °C, similar morphology was only formed and comprised of pure Li_2_Si_2_O_5_ hydrate rods with rectangular cross-sections and length increasing from 2 to 5 µm. Besides, the tops of the Li_2_Si_2_O_5_ hydrate rods were irregular at 120 and 150 °C, whilst they presented square shape at 180 °C (Fig. 2(e)), which showed that increasing the temperature could provide the impetus for the ripening and perfection of rod-like crystals. This was consistent with the XRD results that pure Li_2_Si_2_O_5_ hydrate phase was obtained at different hydrothermal temperatures (Fig. 6(d)) with enhanced peak intensities upon increasing temperatures suitably.

**Figure 7:**
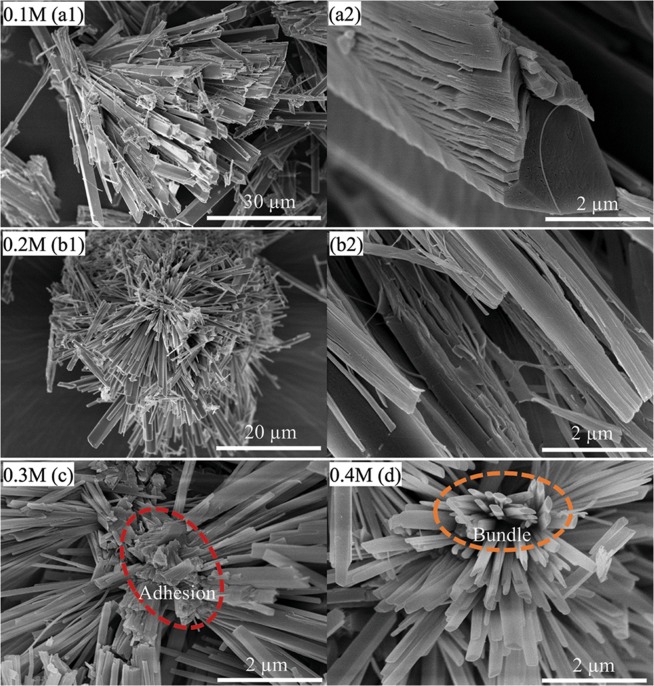
The FE-SEM images of Li_2_Si_2_O_5_ hydrate structures synthesized with different initial precursor concentration at 180 °C for 48 h under Li/Si = 1: 0.1 M (**a1**,**a2**), 0.2 M (**b1**,**b2**), 0.3 M (**c**) and 0.4 M (**d**).

Figure 7 shows the FE-SEM images of Li_2_Si_2_O_5_ hydrate structures synthesized with different initial precursor concentration from 0.1 to 0.4 M at 180 °C for 48 h under Li/Si = 1. As the precursor concentration was 0.1 M, the Li_2_Si_2_O_5_ hydrate rod-like crystals with a width of approx. 2 µm and length of about 40 µm were obtained (Fig. 7(a1)), while the single rod in Fig. 7(a2) had a dehiscent lamellar structure due to the imperfect cleavage caused by the rapid growth. Increasing the precursor concentration to 0.2 M, analogous structures were formed as shown in Fig. 7(b1,b2) except for the width and length of one single rod decreased to about 1 µm and 18 µm, respectively. Compared to the prism-like rods in Fig. 2(e), when the concentrations were 0.3 M and 0.4 M, the dehiscent lamellar structure coalesced and partly disappeared in Fig. 7(c) accompanied by adhesion at the roots of rods, until completely converted to parallelogram cross-sectional rods together with rod-bundles (in Fig. 7(d)). Meanwhile, the size of single rods was dramatically decreased from dozens of micrometers to several micrometers (approx. 7 µm) as the concentration increased from 0.2 to 0.5 M.

Consequently, as indicated, the Li/Si molar ratio, hydrothermal temperature and precursor concentration could have critical effects on the morphology, phase composition and crystal size of the final product. In a comprehensive consideration of good-crystallinity and uniform morphology, the parameters of Li/Si = 1, precursor concentration of 0.5 M, temperature of 180 °C, were the optimal choice to obtain pure 3D Li_2_Si_2_O_5_ hydrate structures with good crystal perfection and appropriate size for applications.

### BET analysis

**Figure 8:**
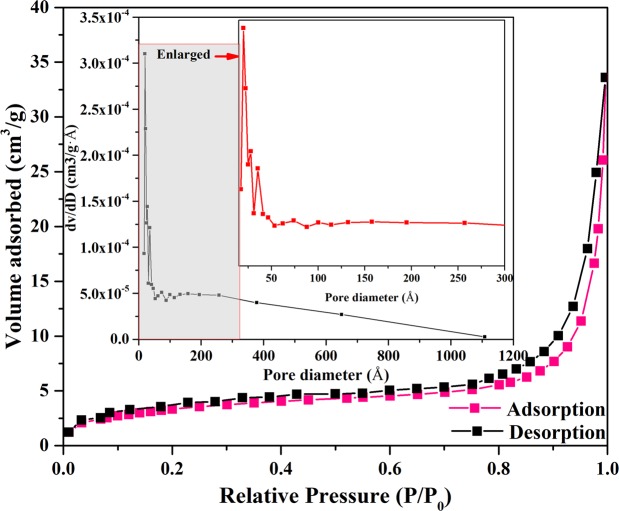
N_2_ adsorption-desorption isotherm and pore-size distribution curve (inset) of the novel 3D Li_2_Si_2_O_5_ hydrate rod assembly.

The N_2_ adsorption-desorption isotherm and pore-size distribution illustrations of the novel 3D Li_2_Si_2_O_5_ hydrate rod assembly (Li/Si = 1, 0.5 M) is shown in Fig. 8. And the corresponding average BET parameters are given in Table 1. It could be observed that the N_2_ adsorption-desorption isotherm featured type IV with a type H3 hysteresis loop^36^ that suggested a mesoporous structure of the 3D Li_2_Si_2_O_5_ hydrate rod assembly with a specific surface area of 18.74 m^2^·g^−1^ (Table 1). This result was also clearly reflected in TEM image (Fig. 2f). The pore-size distribution illustrations indicated the mesopores predominately in the range 2–30 nm with the average pore diameter and pore volume of the 3D structures were 13.92 nm and 4.84 × 10^−2^ cm^3^ g^−1^ (Table 1). Besides, the observed hysteresis loop shifted to a higher relative pressure on *P/P*_0_ ≈ 1, suggesting the presence of macropores (>50 nm) as confirmed by up to 112 nm (pore-size distribution in Fig. 8). The macropores presumably arose from the void space formed by the stacked rods. The mesoporous morphology was likely to benefit adsorption performance.

**Table 1:** BET parameters of 3D Li_2_Si_2_O_5_ hydrate structures.

BET surface area (m^2^·g^−1^)	18.74
Average pore diameter (nm)	13.92
Pore volume (cm^3^·g^−1^)	4.84 × 10^−2^

### Adsorption performance

**Figure 9:**
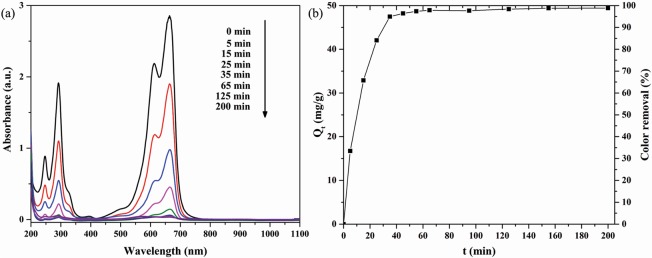
(**a**) The UV-vis spectra and (**b**) adsorption curve of the novel 3D Li_2_Si_2_O_5_ hydrate structures.

To demonstrate the potential applicability of the as-prepared 3D hierarchical Li_2_Si_2_O_5_ hydrate rod assembly (Li/Si = 1, 0.5 M), we investigated their adsorption activity by employing the adsorption of methylene blue at room temperature. The UV-vis spectra and adsorption curve of the novel 3D Li_2_Si_2_O_5_ hydrate structures is shown in Fig. 9. As shown in Fig. 9a, it could be observed that the characteristic peak of methylene blue at 664 nm decreased dramatically in the beginning stage of 5 min and 15 min. This was ascribed to the strong electrostatic interaction between positively charged methylene blue and negatively charged Li_2_Si_2_O_5_ hydrate surfaces in aqueous solution with pH value of about 7^37,38^. All of the vacant surface sites from the mesoporous structure were fully exposed to the methylene blue solution and facilitated the adsorption property. While with a lapse of adsorptive time, the peak decrease of methylene blue slowed down. This was because the remaining vacant surface sites were difficult to be occupied due to the steric barrier between methylene blue molecules adsorbed on the surface and solution^39^. The absorbance of methylene blue almost remained constant from 65 min till 200 min, suggesting the adsorption equilibrium. Figure 9b demonstrated the removal extent of methylene blue solution by the novel 3D Li_2_Si_2_O_5_ hydrate structures. It was apparent that the rate of methylene blue adsorption was drastic in the initial stage, then slowed down and finally constant after equilibrium, which was consistent with UV-vis spectra (Fig. 9a). The equilibrium color removal and adsorption amount of methylene blue were as high as 98.85% and 49.42 mg g^−1^, respectively, showing that the 3D hierarchical Li_2_Si_2_O_5_ hydrate rod assembly had an excellent adsorption activity and could be used in wastewater treatment.

## Conclusions

In our study, novel hierarchical 3D Li_2_Si_2_O_5_ hydrate hollow flower-like microstructures assembled by rods were successfully fabricated by a hydrothermal route using precursors of LiOH·H_2_O and TEOS with Li/Si = 1. The results showed that the anisotropic growth of Li_2_Si_2_O_5_ hydrate benefited from the sustainably high chemical potential attributed to that the metastable phases with poor-crystallinity ensured a certain dissolution rate in the hydrothermal system. The complete Li_2_SiO_3_-to-Li_2_Si_2_O_5_ hydrate transformation indicated the significant importance in selecting precursors that could circumvent the common ion effect in other reports. Meanwhile, the variation of Li/Si molar ratios confirmed that Li_2_Si_2_O_5_ hydrate rods were obtained only at Li/Si = 1. The perfection and aspect ratio of the rods could be controlled very well by adjusting the hydrothermal temperatures and precursor concentrations. The hierarchical 3D hollow microstructures provided a high BET surface area and mesoporous structure that led to an excellent methylene blue adsorption performance. They exhibited extraordinarily high adsorption amount of 49.42 mg g^−1^ and color removal of 98.85%. We believed that the hierarchical 3D hollow structures synthesized using this method had considerable potential application in wastewater treatment.
